# A psychometric investigation of the Chinese version of the Internal, Personal and Situational Attributions Questionnaire (C-IPSAQ)

**DOI:** 10.1038/s41398-018-0314-4

**Published:** 2018-11-28

**Authors:** Bin Gao, Yiquan Wang, Yihong Zhu, Qi Tian, Zhiyu Chen, Zachary Cohen, Yulia Landa, Kim T. Mueser

**Affiliations:** 1 Department of Psychiatry, The Second Affiliated Hospital, Zhejiang University, Hangzhou, Zhejiang 310009 P. R. China; 2 Department of Psychiatry, Hangzhou Seventh People’s Hospital, Hangzhou, Zhejiang 310013 P. R. China; 3 Mental Health Education and Counseling Center of Zhejiang University, Hangzhou, Zhejiang 310058 P. R. China; 4 Department of Public Health, Medical School, Zhejiang University, Hangzhou, Zhejiang 310058 P. R. China; 5 Department of Psychiatry, Beijing nanyuan hospital, Fengtai district Beijing, 100076 P. R. China; 6 Department of Psychiatry, Weill Medical College of Cornell University, New York, NY USA; 7 Department of Psychiatry, Icahn School of Medicine at Mount Sinai, New York, NY USA; 8 Center for Psychiatric Rehabilitation of Boston University, Boston, MA USA

## Abstract

The IPSAQ is a self-administered instrument designed to evaluate individuals’ attributional style (AS). The purpose of this study is to examine the psychometric properties of the Chinese version of the Internal, Personal and Situational Attributions Questionnaire (C-IPSAQ). We also investigate if patients with depression and patients with delusions exhibit attributional biases. The English version of IPSAQ was translated into Chinese and back-translated into English for use in this study. 200 normal control individuals, 47 depressed patients, and 41 delusional patients diagnosed with schizophrenia were recruited for this study. Psychometric properties of this questionnaire were evaluated. The IPSAQ was found to have good internal consistency as a scale. The mean Cronbach’s alpha of the six subscales was 0.697. The inter-rater reliability was also acceptable. The concurrent validity analysis revealed that the C-IPSAQ was significantly correlated with ASQ. The group-comparison analyses showed differences in attributional style between patients with depression and patients with delusions compared to normal controls. We confirmed the reliability and validity of the C-IPSAQ, and that the instrument can discriminate specific attributional biases between different patient populations. The C-IPSAQ is a valid instrument to assess attributional style in delusional and depressed patients.

## Introduction

Social cognition refers to the cognitive processes involved in how people think about themselves, other people, social situations, and interactions^1^. One important element of social cognition is attributional style (AS), which refers to an individual’s habitual way of explaining the causes of positive and negative events in their life^2^.

The role of the AS in symptom formation has been routinely studied. The majority of studies on AS have focused on depression, with a number of studies showing that individuals with depression tended to excessively make internal, stable, and global attributions for negative events^3–7^. This is termed depressive attributional style^8^. The greater tendency to see negative events arising from internal, stable, and global causes is associated with higher levels of depressive symptoms^9^.

Recently, studies have focused more on the AS of patients with delusions. The first study to demonstrate an abnormal attributional style in patients with delusions was reported by Kaney and Bentall^10^. They used Peterson et al.’s Attribution Style Questionnaire (ASQ)^11^ to show that patients with delusions excessively make internal attributions for positive events and external attributions for negative events, a tendency that appears to be an exaggeration of the self-serving bias observed in ordinary people^12^. Fear et al. reported a similar attribution bias in individuals with delusions^13^. Bentall et al. argued that delusional ideation may be a product of abnormal attribution processes^14^. He has proposed an attributional model of delusions asserting that external attributions reduce the accessibility of actual-self/ ideal-self discrepancies, but increase the accessibility of actual-self/ actual-other discrepancies (Actual-self is one’s representation of the attributes that one believes he or she actually possesses. Ideal-self is one’s representation of the attributes that he or she should ideally possess. Actual-other is one’s representation of the attributes that he or she possesses in the eyes of others). Bentall et al. and McKay et al. have also argued that delusions function as a defense to protect the individual against low self-esteem, which might result from the threat to self-concept^15,16^. Blackwood et al. mentioned relevant contents in recent research^17^. Self-serving bias was associated with lower positive and higher negative self-esteem in patients with delusions^18,19^. DeVylder et al. proposed that a biased attributional style is likely not a trait that contributes to emergent paranoid delusions but is instead a state-dependent correlate of paranoid delusions^20^.

Although the ASQ has been broadly used to measure AS, it has been criticized for its poor reliability (specifically the lower reliability of its internality-causal-locus dimension^21,22^ and its unidimensional internal-external domain^23^). To assess all three attribution loci: internal, external-situational and external-personal causes, Kinderman and colleagues developed the Internal, Personal and Situational Attributions Questionnaire (IPSAQ)^24^, and confirmed that the sub-scales of this questionnaire have superior reliability compared to the ASQ. Kinderman’s subsequent study found the presence of an externalizing bias (EB; the greater tendency to make external attributions to negative than positive events) in deluded patients. Moreover, patients with delusions were shown to have an excessive personalizing bias (PB; the tendency to attribute negative events to external-personal as opposed to external-situational causes)^25^. Using the IPSAQ, other studies have reported externalizing attributional bias in people with delusions^26^. Blackwood et al. has also reported that people with persecutory delusions tend to attribute negative events to external-personal causes^17^ and Bental et al. argued that the bias of attributing negative events to external agents contributes to the building of a paranoid worldview^27^. Aakre et al. put forward that therapeutic approaches could help to retrain patients in their ways of explaining life events because of the link between persecutory content and attributions^28^. However, Brakoulias noted that attributional biases are highly variable and appear to relate to an individual’s specific delusional content^29^. For example, McKay and Cipolotti discussed an association of Cotard delusion (a rare neuropsychiatric disorder in which the patient holds a delusional belief that he or she is dead) with an internalizing attributional style^30^. In addition, Diez-Alegria, et al. found that PB varies with the degree of the severity of psychopathology^31^. Mizrahi, et al. also found that patients who tend to use internal attributions for negative events exhibit more severe psychopathology^32^.

In China, previous studies have used the ASQ to explore the relationship between attribution and depression, and have reported similar results^33–35^. However, the association between AS and delusions has never been investigated in China. We therefore sought to study the AS of the Chinese psychotic population. The purpose of this study is to investigate the reliability and validity of the Internal, Personal and Situational Attributions Questionnaire (IPSAQ) for the Chinese population, and to investigate if there are attributional biases in patients with depression and patients with delusions. We hope that such an investigation of the psychometric properties of the IPSAQ in a different cultural setting will broaden the theoretical and empirical basis of the scale, and provide additional knowledge for the versatility of its properties. To our knowledge, there are no documented studies on the psychometric properties of IPSAQ in China. This study may have important clinical implications for investigating AS in Chinese psychotic illness patients.

In addition, in recent years, psychotherapies such as Cognitive Behavioral Therapy (CBT) and Social Cognition Therapy have been applied broadly for schizophrenia patients with delusions as effective, supplementary treatments to medication. One targeted goal of these psychotherapies is the modification of attributional biases^29,36–38^. The patients’ attributional biases contribute to the fixity of their delusional ideations, rendering them impervious to contradictory evidence. The modification of attributional biases can assist in destabilizing the delusional conviction. Psychotherapy has become increasingly popular in treating psychotic disorders in China, and the IPSAQ will be a useful instrument in measuring attributional changes following treatment.

## Methods

### Participants

A cross-sectional study using the translated IPSAQ was conducted across three subgroups of subjects: (1) delusional group (2) depressed group (3) normal control group. The delusional group included 41 patients with the diagnosis of schizophrenia with delusions, aged 18 to 58. Ten patients had previously exhibited symptoms of delusions, but did not exhibit delusions at the time of assessment. Thirty-one patients were currently experiencing delusions at the time of assessment. The depressed group was comprised of 47 patients, aged 14 to 60, with the diagnosis of depressive disorder without psychotic features. All patients were recruited from the same psychiatric hospital in Hangzhou, from both inpatient and outpatient units. The normal control group consisted of 200 normal control subjects, aged 16 to 60; participants included community residents and college students. Individuals with a history of psychiatric illness or a professional background in mental health were excluded. Demographic information for the three samples used in this study is presented in Table 1. All of the patients were diagnosed using the *DSM-IV* by licensed psychiatrists. The Positive and Negative Syndrome Scale (PANSS) was used to assess symptom severity.

**Table 1:** Sociodemographic and illness characteristics of the participants

Characteristics	Group		
	Normal control group	Depressed group	Delusional group
Sample size	200	47	41
Gender (% male)	29.0	46.8	48.8
Education (%)			
Post graduate	6.0	4.3	2.4
College	65.0	40.4	36.6
Senior middle school	18.5	21.3	41.5
Under or junior middle school	10.5	34.0	19.5
Age (Mean ± SD)	20.72 ± 10.77	34.40 ± 12.38	32.00 ± 10.55

This study was approved by the ethics committee of the Seventh People’s Hospital of Hangzhou and Zhejiang University. We gave each participant or the legally authorized representative detailed information on the processes (including possible risks) of the study with commonly used words until that he/she understood completely. When he/she agreed, he/she would sign an informed consent. Written informed consent of the schizophrenic patient was obtained from his/her legally authorized representative and the control provided written informed consent himself/herself.

### Procedures

All study participants completed the Chinese version of the Internal, Personal and Situational Attributions Questionnaire (C-IPSAQ) and the Chinese version of Beck Depression Inventory (C-BDI). In addition, the normal control group also completed the Chinese version of Attribution Style Questionnaire (C-ASQ). Following a comprehensive explanation of this study, all participants were asked to sign a consent form. Questionnaires were distributed by trained research assistants, who answered any questions that arose from participants. Completion of the questionnaires required approximately 45 min for all participants. Ninety-seven percent of the participants completed all of the questionnaires. Six participants did not finish the questionnaires, as they had difficulties in understanding the questions even after an explanation. Two participants declined to complete the questionnaires.

### Measures

#### Internal, Personal and Situational Attributions Questionnaire (IPSAQ)

The IPSAQ is a self-report instrument designed to evaluate attributional style. It is a 32-item questionnaire that includes 16 hypothetical social situations with positive outcomes and 16 with negative outcomes. Positive and negative events are randomly ordered in the questionnaire. Participants are instructed to vividly imagine each situation and write down the one most likely cause for each situation. They are then asked to indicate if the outcome is primarily something due to themselves (internal attribution), something due to others (external-personal attribution), or something due to the situation (external-situational attribution). Scoring involves summing the number of internal, external-personal, and external-situational attributions for positive and negative events separately. EB score is calculated by subtracting the number of internal attributions for negative events from the number of internal attributions for positive events. A positive EB score reflects a tendency to make external attributions for negative events (self-serving bias). A PB score is calculated by dividing the number of personal attributions by the sum of both personal and situational attributions for negative events. A PB score greater than 0.5 represents a tendency to blame others rather than situational factors for negative events. Kinderman and Bentall (Kinderman and Bentall, 1996) reported satisfactory internal reliability for this instrument, with a mean alpha of 0.675^24^.

The forward-backward translation procedures were applied to translate the IPSAQ from English into the Chinese language. The study investigator (Y.Z.) translated the questionnaire into Chinese, which was then back translated into English by a different native Chinese speaker. A native English speaker (Z.C.) then compared the original English version and the back-translated English version. Modification of the Chinese version was based on the result of the comparison. Subsequently, a provisional version of the Chinese questionnaire was developed, and a pilot study was performed with 144 healthy respondents. Small revisions were made to the translated version as a result of the pilot study’s findings. Ultimately, a final C-IPSAQ was used in this study.

#### Attribution Style Questionnaire (ASQ)

The ASQ^11^ is a 12-item scale requiring participants to state the likely cause for each of 6 hypothetical positive and 6 hypothetical negative events, and then rate the cause on three 7-point scales of internality (ranging from totally due to others or totally due to self), globalness (whether the cause would affect other areas of life), and stability (whether the cause was likely to be present in the future). Higher scores represent more internal, global and stable attributions. Scores of internality are obtained by separately summing the responses for positive and negative items. The SSB (self-serving bias) score is calculated by subtracting the internality score for negative events from that for positive events. Although the internal reliabilities of the ASQ subscales are poor, it has been extensively used in psychopathology research since it taps into a domain of considerable psychological importance^39^. The reliability and validity of the C-ASQ were confirmed in a previous study^40^.

#### Beck Depression Inventory (BDI)

The BDI is a 21-item self-report instrument used to measure current depressive symptoms^41^. Respondents rate each item on a 4-point scale according to their mood over the past 7 days. The BDI has been shown to have good reliability and has been used extensively in depression research^39^. Two previous local studies conducted by Shek^42,43^ also confirmed the reliability and validity of the C-BDI.

### Statistics

The analysis was conducted using SPSS for Windows (Version 16.0). The internal consistency reliability was assessed by using Cronbach’s alpha for the IPSAQ and its sub-scales. The inter-rater reliability was determined between the subjects themselves and an observer who did not take part in the study. The concurrent validity of the IPASQ was assessed by examining its relationship with the other three questionnaires employed (correlation and regression analysis). The effect sizes for Spearman correlations can be classified as follows: small for *r* ≥ 0.10, medium for *r* ≥ 0.30 and large for *r* ≥ 0.50. The group-comparison analyses were measured by Independent Sample *t*-Tests conducted on C-IPSAQ sub-scale scores and two composite scores (PB and EB) between different groups.

## Results

### Reliability

Reliability statistics (Cronbach’s alpha) revealed acceptable levels of internal reliability for all six subscales and two composite scores: EB and PB. The mean Cronbach’s alpha of the six subscales is 0.697 (range from 0.674 to 0.738) (Table 2). EB and PB were unrelated to one another, Spearman’s *r* *=* 0.036, *p* = 0.586. The causes written by participants were also rated by an observer. Self-rated and observer-rated scores were significantly correlated with each other (Table 3).

**Table 2:** Internal reliability of the C-IPSAQ subscales (Normal controls, *N* = 200)

	PI	PP	PS	NI	NP	NS	PB	EB
Mean	8.67	3.57	3.74	6.24	5.49	4.140	0.57	2.43
SD	3.09	2.63	2.67	3.13	3.03	3.191	0.28	4.14
Cronbach’s *α*	0.712	0.679	0.687	0.690	0.674	0.738		

*PI* internal attribution for positive events, *PP* personal attribution for positive events, *PS* situational attribution for positive events, *NI* internal attribution for negative events, *NP* personal attribution for negative events, *NS* situational attribution for negative events, *EB* externalizing bias, *PB* personalizing bias

**Table 3:** The two-tailed Spearman correlations of self and observer-rated scores (inter-rater reliability) (Normal controls, *N* = 110)

	PI	PP	PS	NI	NP	NS	EB	PB
Spearman’s *r*	.573	.582	.611	.796	.724	.564	.797	.540
*p*	.000	.000	.000	.000	.000	.000	.000	.000

### Validity

The concurrent validity of the C-IPASQ was assessed by examining its relationship with the other questionnaires employed. The correlations of C-IPSAQ and C-ASQ are presented in Table 4. EB was significantly correlated with SSB score of C-ASQ. NI (internal attribution for negative events) subscale score was significantly correlated with C-ASQ negative-internality subscale score. PI (internal attribution for positive events) was significantly correlated with Positive-internality subscale of C-ASQ. EB correlated with C-BDI scores, spearman’s *r* = − 0.195, *p* = 0.006. Regression analysis also revealed that EB was predicted by C-BDI scores, *β* = −0.143, *t* = −2.017, *p* = 0.045.

**Table 4:** The two-tailed Spearman correlations of C-IPSAQ and C-ASQ (Normal controls, *N* = 200)

	EB	PI	NI
SSB	0.286**		
Positive-internality		0.154^*^	
Negative-internality			0.226**

^*^p < 0.05, **p < 0.01

Group-comparison analysis was used to examine whether the C-IPSAQ could discriminate between different groups. We allocated all of the normal control participants into two groups based solely on BDI score. Based on the BDI cutoff criterion of symptom score of less than five (less than 5 indicates no depression)^44^, Group 1 was comprised of 78 participants who had BDI score less than 5. Group 2 was comprised of 120 participants who had BDI score greater than or equal to 5. Two participants who didn’t finish BDI scales were excluded. An Independent-Sample *t*-Test conducted on EB score revealed significant differences between groups. The group with higher BDI scores had lower EB scores than the group with lower BDI scores (see Table 5). Individuals with high BDI scores tended to attribute the negative events to themselves, rather than to other people, which suggested a lower self-serving bias.

**Table 5:** Independent *t* Test conducted on EB score of C-IPSAQ in normal controls

Group	*N*	Mean ± SD	*t*	*p*
BDI < 5	78	1.82 ± 3.93	−2.692	0.008
BDI ≥ 5	120	3.42 ± 4.30		

For the delusional group we also conducted an independent *t*-Test on IPSAQ subscales and EB and PB scores between the higher and lower BDI score groups (based on a BDI cutoff score of 5). There were no differences between the two groups. The above results indicate that in the normal control group, individuals’ attributional style was associated with BDI scores. Thus, adjustments were made before comparing the attributional style of both patient groups and the normal control group separately. Based on the BDI cutoff criterion of a symptom score of 5 (less than 5 means “no depression”), 78 participants whose BDI scores were less than a cutoff of 5 from the Control group were included in Group C’. Group D’ only comprised 43 participants from the depressed group whose BDI scores were 5 or greater. Thirty-one patients who were currently experiencing delusions from delusional group were included in Group P’. An Independent-Sample *t*-Test conducted on NS revealed significant differences between Group C’ and Group P’. Group P’ made less situational attributions to negative events than did Group C’. Other sub-scores, EB, and PB did not vary across the groups. An Independent-Sample *t*-Test conducted on EB revealed significant differences between Group C’ and Group D’ (Table 6).

**Table 6:** An independent-sample *t* test conducted on C-IPSAQ subscales for selected groups

	Group C’	Group D’	Group P’		
	*N* = 78	*N* = 43	*N* = 31	*t*	*p*
	(Mean ± SD)	(Mean ± SD)	(Mean ± SD)		
EB	3.42 ± 4.31	1.67 ± 4.43		2.011	0.037
NS	4.32 ± 3.24		3.06 ± 2.41	2.175	<0.05

The patients in the Delusional Group who did not exhibit delusional symptoms w*e*re given a score of 1 (absent) for Item P1 (delusions) on the Positive and Negative scale of the Positive Syndrome Scales (PANSS)^45^. An item P1 score of at least 2 or greater indicates delusional ideation. Thus, item P1 = 2 was used as a cutoff point value^22^. An Independent *t-*Test conducted on PB scores revealed a significant difference between patients with higher and lower P1 scores. Also using P1 = 2 as a cut point value, an Independent *t*-Test conducted on NS scores revealed a significant difference between patients with higher and lower P1 scores (Table 7). The patients experiencing delusions (P1 > = 2) showed lower NS and higher PB scores than remitted patients not experiencing delusions (P1 = 1), respectively.

**Table 7:** An Independent t Test conducted on C-IPSAQ subscales in Delusional. Group

	Group(P1 = 1)	Group(P1 > = 2)		
	N = 10	N = 31	t	p
	(Mean ± SD)	(Mean ± SD)		
NS	6.50 ± 3.78	3.06 ± 2.41	3.393	0.002
PB	0.39 ± 0.31	0.67 ± 0.28	2.699	0.010

## Discussion

The objective of this study was to investigate the reliability and validity of the C-IPSAQ in the Chinese population. The results of the reliability analyses indicate that the Chinese version of IPSAQ is reliable in terms of Cronbach’s alpha. The reliabilities of C-IPSAQ subscales (mean alpha = 0.697) are superior to that of the original English version of the IPSAQ, as reported by Kinderman (mean alpha = 0.675). Its inter-rater reliability is also satisfactory. The correlations of the C-ASQ with the C-IPSAQ suggest that they are measuring the same psychological construct. The C-IPSAQ’s EB score, PI subscale and C-ASQ’s SSB score, Positive-internality subscale appear to be similar in measuring self-serving bias. The C-IPSAQ’s NI subscale appears to be analogous to the C-ASQ negative-internality subscale in measuring the bias of self-blame.

The group-comparison analyses showed that the C-IPSAQ can discriminate specific attributional biases between different patient populations. We found that (1) people with depression seem more likely to attribute negative events to internal causes; (2) compared to normal controls, patients who experience delusions make less external-situational attributions for negative events; (3) patients who are experiencing delusions make fewer external-situational but more external-personal attributions to negative events than schizophrenia patients who are not experiencing delusions.

The correlation and regression analysis of EB and BDI scores suggest that people with high BDI scores are more likely to attribute negative events to internal causes than personal or situational ones. These findings are consistent with the results of previous studies^3,5–7^. However, we did not find the same results in patients with delusions; mood seemed to have no effect on AS for these patients. This may suggest that patients with delusions have some specific AS that is different from normal controls. The results of Independent-Sample *t-*Tests conducted on EB also suggest that depressed patients exhibit relatively less of a self-serving bias, which is consistent with the results of Kaney, et al.’s study^46^.

Compared to normal controls, patients who experience delusions made less external-situational attributions for negative events. Kinderman and Bental had suggested that external-situational attribution for negative events appeared to be psychologically benign^24^. Contrary to Kinderman et al^25^., we did not find that patients with delusions made more external-personal attributions to negative events than normal controls. That could be explained by cultural differences, as individuals who grew up in different cultures may have different thinking styles or habits that shape their cognitive processes. It has been found that East Asians, on average, generate more situational inferences than Westerners when making attributions^47,48^. This may be why we only found differences in situational attributions, but did not find differences in external-personal attributions between deluded patients and normal controls. We also found significant differences in NS and PB between patients who were experiencing delusions and those who were not. Delusional patients made less external-situational but more external-personal attributions to negative events comparing to patients who did not hold delusional beliefs. This may suggest that attributional biases are unstable, and PB might be a rather unspecific characteristic that varies with the degree of the delusion’s severity. This finding is similar to that of Diez-Alegria et al.’s study^31^. The patients who were experiencing delusions showed more personalizing bias than the patients who were not, suggesting that the magnitude of the bias was related to the patient’s clinical state.

In our study, not only do all of the observations across the group-comparisons confirm the validity of the C-IPSAQ, but they also have implications for the general understanding of AS in Chinese patients with depression or delusions. Due to the poor reliability of C-ASQ, there was little investigation of AS in Chinese psychotic patients. We have found that the reliability of C-IPSAQ is higher than C-ASQ, and that it can discriminate different AS between psychotic patients and normal controls. From a clinical perspective, AS can be used as an index in measuring the effect of some psychotherapies, such as CBT, Social Cognition Therapy, and others for patients with delusions. IPSAQ has been used as an instrument to indicate the reasoning changes achieved when applying CBT to treat people with delusions^29,49^. In this study, AS varied based on the severity of delusions in Chinese patients. It would be important to further test whether changes in attributional biases mediate delusional conviction. In view of the growing interest in applying psychotherapies such as CBT for Chinese people with delusions, this Chinese version of IPSAQ may be used to study mechanisms of change in CBT for delusions that are specific to Chinese patient population.

This study does have some limitations. First, our sample was restricted. We did not use randomly chosen subjects as uncooperative subjects were not included. Secondly, we did not classify the content of the delusions even though it was found that attributional biases appeared to relate to an individual’s specific delusional content^29^. Another limitation of this study is that we did not investigate the delusion proneness of normal participants. The delusion proneness in normal individuals has been reported in several previous studies. Therefore, it may have affected our findings on the correlations between attributional style and delusions. The investigation of the delusion proneness in the normal control population is necessary in future studies.

In conclusions, the present study confirmed the reliability and validity of the Chinese version of IPSAQ among Chinese adults, and sought to investigate the differences in attributional style among different samples of Chinese people. The results indicated that the Chinese version of IPSAQ is an instrument with adequate psychometric properties that can assess attributional style. The C-IPSAQ may serve as a useful tool to determine attributional bias in patients with depression and patients with delusions.
